# Nutritional assessment and post-procedural complications in older stroke patients after insertion of percutaneous endoscopic gastrostomy – a retrospective study

**DOI:** 10.3402/fnr.v60.30456

**Published:** 2016-08-02

**Authors:** Gunnel Wärn Hede, Gerd Faxén-Irving, Ann Ödlund Olin, Britt Ebbeskog, Milita Crisby

**Affiliations:** 1 Division of Nursing, Department of Neurobiology, Care Sciences and Society, Karolinska Institutet, Stockholm, Sweden; 2 Division of Clinical Geriatrics, Department of Neurobiology, Care Sciences and Society, Karolinska Institutet, Stockholm, Sweden; 3 Clinical Nutrition and Dietetics, Karolinska University Hospital, Stockholm, Sweden; 4 Department of Quality and Patient Safety, Karolinska University Hospital, Stockholm, Sweden

**Keywords:** stroke, oropharyngeal dysphagia, percutaneous endoscopic gastrostomy, nutritional assessment, post-procedural complications

## Abstract

**Background:**

Oropharyngeal dysphagia is one of the major complications of stroke and a risk factor for malnutrition and prolonged in-hospital stay.

**Objective:**

The overall aim was to describe to what extent nutritional assessments (i.e. BMI kg/m^2^, eating problem, and weight loss) were performed and documented in the records of older stroke patients treated with enteral nutrition by percutaneous endoscopic gastrostomy (PEG). A secondary aim was to identify documented post-procedural complications after PEG insertion during hospital stay.

**Design:**

The study is retrospective. Data were collected from records of 161 stroke patients ≥65 years, who received PEG, admitted to three stroke units during a 4-year period.

**Results:**

Mean age of the patients was 82.2 (±7) years, and 86% of the patients were ≥75 years old. On admission, body weight was documented in 50% of the patients and at discharge in 38% of the patients. BMI data were not documented at all at discharge in one of the units. Almost 80% of the patients fulfilled the European Network criteria for multimorbidity. Morbidity and multimorbidity correlated to the length of stay (*p<*0.0005). Complications were reported in 111 (69%) of the patient records. In 53 patients (33%) more than one complication was reported. A total of 116 pressure ulcers were reported and 30 patients had more than one pressure ulcer. The number of complications was related to weight loss (*p=*0.046) and BMI change (*p=*0.018).

**Conclusions:**

Essential information of the patient's nutritional status was poorly recorded which could affect the patient's nutritional treatment during the hospital stay. This study indicates that implementation of guidelines in patients with stroke is needed. The high number of pressure ulcers was an unexpected finding.

One of the major complications of stroke is oropharyngeal dysphagia (OD), affecting 37–78% of the patients (1). Despite spontaneous recovery, studies have shown that approximately 15% of the stroke patients have persistent swallowing difficulties at 1 month after the acute event (2). OD is a major cause of aspiration pneumonia (1). Furthermore, OD may contribute to poor nutritional status and if not recognized could lead to malnutrition. Malnutrition could in turn counteract clinical recovery and promotes prolonged in-hospital stay as well as increased healthcare costs (3–7). These consequences could be deleterious for stroke patients since malnutrition is a risk factor that promotes infections and affects the quality of life (8). The National Board of Health and Welfare and national guidelines for treatment of stroke patients recommend early nutritional screening (9, 10). These guidelines state that risk factors for malnutrition, that is, unintentional weight loss, eating difficulties, and underweight (BMI<22 kg/m^2^ in patients >70 years), should be recognized early during the course of the hospital stay.

When oral intake is no longer possible due to OD, early initiation of enteral nutrition (EN) is recommended and when a patient needs EN for a longer period of time, nutrition by percutaneous endoscopic gastrostomy (PEG) is preferable to nasogastric tube feeding (11, 12).

Complications due to PEG insertion vary between different studies. Early post-procedural complications occurring within 48 h up to 30 days after PEG insertion are reported in 2–38% patients (13–16). The most common complications in this period are wound infections, fever, local pain, peristomal leakage, diarrhea, constipation, aspiration pneumonia, peritonitis, and mortality (13–16). Late complications occurring 72 h up to 1 year after PEG insertion are reported in 20–28% of the patients (13–16).

The overall aim of this study was to describe to what extent nutritional assessments were performed and documented in the records of older patients with stroke treated with EN by PEG. A secondary aim was to identify documented post-procedural complications after PEG insertion during hospital stay.

## Methods

This is a descriptive study. Data were retrospectively collected from the records of stroke patients ≥65 years old admitted to three stroke units during January 2006 to December 2009. To get a sufficient number of patient records, the data were collected from both the stroke unit at the Department of Neurology (DN) and the stroke unit at the Department of Geriatric Medicine (DG) of Karolinska University Hospital. In addition, data were collected from the stroke unit at the Department of Internal Medicine (DM) at the South hospital in Stockholm, Sweden. The results are presented separately, as the patients’ characteristics in the various units differ with regard to the length of stay (LOS) and their age. Data collection started in October 2011 and ended in January 2012. The stroke diagnoses were defined according to the International Classification of Diseases, 10th edition (ICD-10).

### Data collection

The following data were collected:

*Patient characteristics*: age, gender, LOS, indication for PEG, and primary (the referral diagnosis on admission) and secondary diagnoses.

*Nutritional status*: height (cm), body weight (kg), body mass index (BMI kg/m^2^), weight loss (prior to admission), and eating difficulties. In this study, we report weight loss during hospital stay (=weight at discharge–weight at admission).

*Multimorbidity definition*: The European General Practice Research Network definition for multimorbidity was used which states that ‘Multimorbidity is defined as any combination of chronic disease with at least one other disease (acute or chronic) or biopsychosocial factor (associated or not) or somatic risk factor’ (17).

*Post-procedural complications*: Complications during hospital stay such as leakage, bleeding from stoma, local inflammation, PEG self-extubated, ileus, PEG in abdomen, wound complication, reflux in PEG, PEG-tube blockage, and local abscess were denoted as PEG-related complications. Diarrhea, constipation, and nausea/vomiting were denoted gastrointestinal-related complications.

*Pressure ulcers*: For category description of pressure ulcers (PUs), the International European Pressure Ulcer Advisory Panel's PU classification system was used – Category I: nonblanchable erythema; Category II: partial thickness skin loss; Category III: full thickness skin loss; and Category IV: full thickness tissue loss (18).

### Statistics and ethics

The results are presented as mean (±SD) except LOS and number of days from hospital admission to PEG insertion which are presented as median (range). For correlation analyses, Spearman correlation coefficients were calculated. Statistic evaluations were made using the Statistica software package (Statsoft Inc., Tulsa, OK, USA). The study was approved by the regional ethical committee at Karolinska Institutet in Stockholm, Sweden: 2010/950-31/1. The study was performed according to the Helsinki declaration guidelines.

## Results

### Patient characteristics

Data from a total of 161 patients ≥65 years of age receiving PEG due to OD after stroke were retrieved. The mean age of the patients was 82.2 (±7.03) years and 86% of the patients were ≥75 years old (Table 1). Median number (range) of days from hospital admission to PEG insertion for all patients was 19 (1–187) days, 25 (13–88) in DG, 22 (1–187) in DN, and 14 (2–38) in DM (Table 1). On admission, 75% of the patients lived in their own accommodations. At discharge an identical number of patients were transferred to nursing homes. The LOS was more than 1 month in DG and DN compared to around 3 weeks in DM (Table 1). Age correlated negatively with weight on admission (rho=−0.26, *p=*0.020).

**Table 1:** Characteristics of stroke patients receiving PEG

	DG *n*=25	DN *n*=68	DM *n*=68	All *n*=161
Age (years), mean±SD	85.3±6.5	80.2±7.0	83.1±6.7	82.2±7.0
65–79 years, *n* (%)	5 (20)	31 (46)	21 (31)	57 (35)
≥80 years, *n* (%)	20 (80)	37 (54)	47 (69)	104 (65)
Gender, female (%)	13 (52)	42 (62)	37 (54)	92 (57)
Accommodation on admission				
Own accommodation, *n* (%)	19 (76)	48 (71)	53 (78)	120 (75)
Nursing home, *n* (%)	6 (24)	20 (29)	14 (21)	40 (23)
No information, *n* (%)	0	0	1 (2)	1 (2)
Accommodation at discharge				
Own accommodation, *n* (%)	3 (12)	3 (4)	1 (1)	7 (4)
Nursing home, *n* (%)	19 (76)	53 (78)	48 (71)	120 (75)
Geriatric clinic, *n* (%)	0	7 (10)	17 (25)	24 (15)
Deceased, *n* (%)	3 (12)	4 (6)	2 (3)	9 (6)
No information	0	1 (2)	0	0
Length of stay, median (range)	40 (21–113)	36 (12–216)	21 (8–53)	30 (8–216)
Days from hospital admission to PEG insertion, median (range)	25 (13–88)	22 (1–187)	14 (2–38)	19 (1–187)

Data from three different wards collected from patient records.

DG=Department of Geriatric Medicine, stroke rehab unit. DN=Department of Neurology, stroke unit. DM=Department of Internal Medicine, stroke unit.

### Nutritional assessment

As presented in Table 2, the mean BMI (m/kg^2^) was 23 (±3.8) in the whole group of patients, on admission. However, in DG, the BMI was lower, that is, 19.4 (±1.6) in patients aged >80 years and 21.9 (±3.5) in patients aged 65–79 years which decreased during the hospital stay. A weight loss of 2.3 kg (±3.7) in DG and 1.9 kg (±3.3) in DN was recorded during the hospital stay.

**Table 2:** Body weight (kg) and BMI (kg/m^2^) on admission and at discharge in stroke patients from three different wards

	DG *n*=25	DN *n*=68	DM *n*=68	All *n*=161
Weight on admission				
65–79 years, mean±SD, (*n*)	54.1±8.7 (5)	74.2±14.7 (14)	62.2±26.5 (8)	
≥80 years, mean±SD, (*n*)	60.5±13.5 (18)	63.5±10.7 (21)	64.8±12.2 (15)	
All, mean±SD, (*n*)				64.2±14.8 (81)
Weight at discharge				
65–79 years, mean±SD, (*n*)	55.0±6.5 (4)	69.5±14.7 (18)	–	
≥80 years, mean±SD, (*n*)	58.3±12.5 (18)	65.6±14.1 (21)	56.0±0 (1)	
All, mean±SD, (*n*)				63.7±14.0 (62)
BMI on admission				
65–79 years, mean±SD, (*n*)	19.4±1.6 (4)	25.7±4.7 (10)	24.6±1.8 (6)	
≥80 years, mean±SD, (*n*)	21.9±3.5 (18)	23.8±3.9 (13)	22.8±3.0 (14)	
All, mean±SD, (*n*)				23.2±3.8 (65)
BMI at discharge				
65–79 years, mean±SD, (*n*)	18.6±2.0 (4)	25.3±4.1 (10)	–	
≥80 years, mean±SD, (*n*)	21.2±3.6 (18)	23.7±4.2 (18)	–	
All, mean±SD, (*n*)				22.7±4.2 (50)
Weight change during stay			–	
65–79 years, mean±SD, (*n*)	−2.4±3.8 (4)	−1.9±3.0 (11)		
≥80 years, mean±SD, (*n*)	−2.2±3.8 (18)	−1.9±3.5 (14)	–	
All, mean±SD, (*n*)				−2.0±3.4 (47)
BMI change during stay				
65–79 years, mean±SD, (*n*)	−0.8±1.2 (4)	−0.7±1.1 (8)	–	
≥80 years, mean±SD, (*n*)	−0.7±1.2 (18)	−0.6±1.4 (12)	–	
All, mean±SD, (*n*)				−0.7±1.2 (42)

Data collected from patient records.

DG=Department of Geriatric Medicine, stroke rehab unit. DN=Department of Neurology, stroke unit. DM=Department of Internal Medicine, stroke unit.

Body weight was assessed on admission in 50% of the patients at all three stroke units (92% at DG, 51% at DN, and 34% at DM). At discharge, body weight was assessed in 38% of the patients altogether (88% at DG, 57% at DN, 1.5% at DM). BMI was assessed both on admission and at discharge in 29% of the patients. None of the patients at DM had a documented BMI at discharge. Eating difficulties were reported in most of the patients and OD was stated as the indication for PEG.

### Morbidity and multimorbidity

The most prevalent diagnosis was ischemic stroke which was reported in 88% of the patients. The remaining patients (12%) suffered from hemorrhagic stroke. Seventy-five percent had concomitant cardiovascular diseases and 20% of the patients had type II diabetes mellitus. Almost 80% of the patients fulfilled the European Network criteria for multimorbidity and 58% of the patients had four or more (4–10) diagnoses. Infections (i.e. pneumonia, septicemia, erysipelas, and urinary tract infection (UTI)) during the hospital stay were reported in 82 (51%) of the patients. Thirty-six percent (*n=*58) of the patients were diagnosed with pneumonia and of these 48 (30%) patients developed pneumonia during the hospital stay. Aspiration was identified to be the major cause of pneumonia. The number of diseases and multimorbidity correlated with the LOS (rho=0.37, *p<*0.005). Morbidity was negatively related to weight loss at discharge (rho=−0.30, *p=*0.019) and to BMI at discharge (rho=−0.40, *p=*0.004).

### Post-procedural complications

After PEG insertion, post-procedural complications were reported in 111 (69%) of the patient records. In 53 patients (33%) more than one complication was reported. Complications occurred more frequently in patients >80 years of age. Table 3 displays PEG-related complications and gastrointestinal complications. PEG-related complications were identified as complications due to PEG insertion and gastrointestinal complications were identified as complications due to the EN treatment. The patients at DG and DN experienced local pain to a higher extent than the patients admitted to DM.

**Table 3:** Post-procedural complications and pressure ulcers after PEG insertion in patients, age groups 65–79 years and ≥80 years, from three different wards

	DG *n*=25	DN *n*=**68	DM *n*=**68	All *n*=161
PEG related^a^				
65–79 years, *n* (%)	4 (16)	10 (15)	5 (7)	19 (12)
≥80 years, *n* (%)	7 (28)	11 (16)	11 (16)	29 (18)
Gastrointestinal related^b^				
65–79 years, *n* (%)	1 (4)	9 (13)	3 (4)	13 (8)
≥80 years, *n* (%)	5 (20)	5 (7)	2 (3)	12 (7)
Pain, local				
65–79 years, *n* (%)	2 (8)	10 (15)	4 (6)	16 (10)
≥80 years, *n* (%)	5 (20)	7 (10)	6 (9)	18 (11)
Pressure ulcer				
65–79 years, *n* (%)	5 (20)	17 (25)	6 (9)	28 (17)
≥80 years, *n* (%)	14 (56)	18 (26)	21 (31)	53 (33)

Data collected from patient records.

DG=Department of Geriatric Medicine, stroke rehab unit. DN=Department of Neurology, stroke unit. DM=Department of Internal Medicine, stroke unit.

a PEG-related complications, that is, leakage, bleeding from stoma, local inflammation, PEG self-extubated, ileus, PEG in abdomen, wound complication, reflux in PEG, PEG-tube blockage, and local abscess.

b Gastrointestinal complications, that is, diarrhea, constipation, and nausea/vomiting.

### Pressure ulcers

PUs were documented in 50% of the patients and were more frequent in male patients. A total of 116 PUs was reported. Thirty patients had more than one PU and one patient had PU within all categories, I–IV. The most common PU categories, Categories I and II, were more frequent in patients aged ≥80 years (Table 3). A total of 75 patients developed PU in Category I. Of these, 49 patients were aged ≥80 years and 35 patients developed PU in Category II whereof 19 were aged ≥80 years. The number of complications was related to weight loss (rho=−0.33, *p=*0.046) and BMI change (rho=−0.42, *p=*0.018).

## Discussion

In this retrospective study of collected data from older stroke patients that underwent PEG, insertion due to OD weight loss was a common finding. In two out of the three stroke units, nutritional status appeared to be insufficiently assessed and recorded. The most common PEG-related and gastrointestinal complications reported in the study population were local pain, diarrhea, nausea/vomiting, leakage, and local bleeding. In addition, PU was reported in 50% of the patients.

OD is identified as a major contributor to malnutrition according to a resolution of the Council of Europe (19) and some other investigators (20–22). Therefore, it is impertinent to identify patients who are at risk of malnutrition and to initiate appropriate nutritional treatment. The patients in this retrospective study had at least one risk factor for malnutrition, that is, OD. Moreover, unintentional weight loss was recorded in the two units where weight was recorded at discharge. Mean weight loss was higher in patients at DG in comparison with patients staying at DN. This may be due to the fact that the patients in DG were older and had more comorbidities. Underweight, that is, BMI<22 kg/m^2^ was recorded in both DG and DN patients but not in DM. Insufficient documentation of the patients’ nutritional status increases the risk of malnutrition and complications. It is obvious that adherence to national guidelines for nutritional screening/assessment differed a lot between the wards. Similar findings of insufficient documentation are reported in two Swedish studies regarding stroke care (23, 24). At the department of DG, a comprehensive work to implement the national guidelines (9) has been performed during the past decade which may explain the differences between the units in this study. Poor documentation affects the patient's safety and the continuity of care which could cause delays in giving proper treatment and a high standard of care (25, 26). Since 2015, the Swedish National Board of Health and Welfare states that every caregiver is obliged to have a nutrition care plan including assessment of the patients’ nutritional status, prevention and treatment of malnutrition (27).

The fact that the number of days from hospital admission to PEG insertion was 19 (1–187) and highest in DG (25 days) may to some extent explain both weight loss and the high prevalence of PUs. PUs occurred in 50% of the patients during hospital stay which is an unexpected finding. PUs were more common in patients staying at DN and DM than in patients at DG although the patients in this unit had the longest LOS. The concomitant findings of PU and malnutrition could result in poor outcome and recovery. Additional risk factors of importance for PU are activity or mobility limitations and skin status (18). One study in severely disabled patients after stroke reported a prevalence of 22% PUs (28). A study from Scotland stated that PU occurred in 21% of the stroke patients and that it was most frequent during acute hospital stay (29). A lack of reporting or poor documentation could be a cause of the discrepancies between the lower frequencies of PU reported in those two studies compared with the present.

International guidelines for prevention of PU state that early and repeated risk assessment is crucial. A structured risk assessment should be followed by a plan for PU prevention if the patient is at risk (18). Despite these guidelines, PUs in hospitalized patients are reported to be common (30) and an important predictor of prolonged hospital stay in older patients (31). As reported in the results of this study, weight change, BMI change, and age were identified as major risk factors for overall complications. Other risk factors for developing PUs are cardiovascular diseases, type II diabetes mellitus, infections, acute illness, age, and general health status (32).

### Limitations

The sample of the included records in this study was small but represented all patients that received PEG during the 4-year period. The small sample constitutes a limitation for the interpretation of the study results due to insufficient documentation of the patients’ nutritional status.

## Conclusions

This study of old stroke patients with OD receiving PEG illustrates that essential information of the patient's nutritional status was missing according to the documentation retrieved from the patient's records. The high number of days from hospital admission to PEG insertion may to some extent explain the weight loss during the hospital stay. Both PEG-related and gastrointestinal-related complications occurred after inserting PEG. PUs were an unexpected finding and occurred in 50% of the patients. The present study indicates that more effective implementation of guidelines in patients with stroke is necessary.
